# Increasing Trends of Herpes Zoster in Australia

**DOI:** 10.1371/journal.pone.0125025

**Published:** 2015-04-30

**Authors:** Raina MacIntyre, Alicia Stein, Christopher Harrison, Helena Britt, Abela Mahimbo, Anthony Cunningham

**Affiliations:** 1 School of Public Health and Community Medicine, University of New South Wales, Sydney, Australia; 2 National Centre for Immunisation Research (NCIRS), Sydney, Australia; 3 bioCSL, Melbourne, Australia; 4 Family Medicine Research Centre, University of Sydney, Sydney, Australia; 5 Westmead Millennium Institute, University of Sydney, Sydney, Australia; University of Cincinnati School of Medicine, UNITED STATES

## Abstract

**Background:**

Increasing trends in incidence of herpes zoster (HZ) have been reported in Australia and internationally. This may reflect the impact of childhood VZV vaccination programs introduced universally in Australia in late 2005. The objective of this study was to evaluate changes in incidence of HZ and PHN in Australia over time, and associated healthcare resource utilisation.

**Methods:**

Australian data on general practice (GP) encounters for HZ, specific antiviral prescribing data from the pharmaceutical benefits scheme, emergency department presentations from the states of NSW and Victoria and national hospitalisation data for HZ were analysed for time trends using regression models. Two time periods (2000-2006 and 2006-2013) were compared which correspond broadly with the pre- and post- universal VZV vaccination period.

**Results:**

All data sources showed increasing rates of HZ with age and over time. The GP database showed a significant annual increase in encounters for HZ of 2.5 per 100,000 between 1998 and 2013, and the rates of prescriptions for HZ increased by 4.2% per year between 2002 and 2012. In the 60+ population HZ incidence was estimated to increase from 11.9 to 15.4 per 1,000 persons using GP data or from 12.8 to 14.2 per 1,000 persons using prescription data (p<0.05, between the two periods). Hospitalisation data did not show the same increasing trend over time, except for the age group ≥80 years. Most emergency visits for HZ were not admitted, and showed significant increases over time.

**Discussion:**

The burden of HZ in Australia is substantial, and continues to increase over time. This increase is seen both pre- and post-universal VZV vaccination in 2005, and is most prominent in the older population. The substantial burden of HZ, along with ageing of the Australian population and the importance of healthy ageing, warrants consideration of HZ vaccination for the elderly.

## Introduction

Herpes zoster (HZ) is a potentially debilitating neurotropic disease caused by neural reactivation of latent varicella zoster virus which causes chicken pox (varicella) as a primary infection [1]. The incidence of HZ rises rapidly after the age of 50, correlated with a decline in cell mediated immunity, and is also high in people with immunosuppressive conditions [1,2]. Among adults aged 22 years and over, approximately 70% of HZ cases occur after 50 years of age; and approximately half of those who reach 85 years will have suffered at least one episode of HZ [3–5]. We have previously described the age-dependent incidence of HZ in Australia [2]. Over the period 2000–2006 incidence was estimated as 2.5 and 10 per 1,000 persons respectively for the age groups below 50 years and 50 years and older Other studies around the world describe similar rates [6]. Post-herpetic neuralgia (PHN) is the most common debilitating complication of HZ, and is a major contributor to the associated morbidity [7,8]. PHN occurs in a third of hospitalised HZ cases, and in immunocompetent adults predominantly occurs in those over 50 years and increases with age [8,9]. The management of HZ and its complications impose a significant social and economic burden on not only the communities affected but also the health system in general [10]. In Australia, the annual costs directly related to HZ for persons aged 50 years and older were approximately $33 million in 2006 [2].

The burden of HZ in Australia is proportionately greater than that of varicella [2,11–14]. HZ is associated with more hospitalisations than varicella; and HZ cases are more likely to be diagnosed with complications than varicella, with neurological complications in approximately a third of hospitalised cases [11].

An outstanding question is whether the epidemiology of HZ is changing over time. The incidence of HZ will be influenced by the proportion of the population that have had VZV infection in the past and by factors that influence reactivation. Hope-Simpson hypothesized that exposure to varicella naturally boosts immunity to the zoster virus thereby preventing reactivation of HZ [15]. Based on this, modeling suggests that in countries with universal varicella immunization, the reduced exposure to wild-type varicella may result in an increased incidence of HZ for 30–50 years post program implementation [16–20]. A trend of increasing HZ incidence has been reported in countries such as the US and Australia that have implemented universal childhood VZV vaccination, but mostly these trends precede the introduction of universal varicella vaccination, making it difficult to determine the contribution, if any, of varicella vaccination to the trend [12,21–24].

In Australia, VZV vaccines for children from 12 months of age became available in 2000 and a universal childhood VZV vaccination at 18 months was implemented in November 2005 [25]. More recently a vaccine to prevent HZ and PHN has become available. This live attenuated Oka-strain HZ vaccine is 14 times more potent than varicella vaccine, and is available and recommended for immunocompetent adults aged 50 years and over [26]. The Shingles Prevention Study (SPS) showed this vaccine to have efficacy against HZ, PHN and a burden of HZ measure of 51%, 66.5% and 61% respectively over 3 years of follow-up [27]. Population based effectiveness studies have confirmed the vaccine reduces the incidence of HZ and PHN among older adults [28,29]. A sub-study of the SPS found evidence of waning efficacy over time with overall efficacy 7 years post vaccination of 40%, 60% and 50% against HZ, PHN and burden of zoster respectively [30].

Given the established universal varicella vaccination program in Australia and the availability of a HZ vaccine, it is therefore timely and important to evaluate changes in the burden of HZ. To approximate the pre- and post-varicella vaccination period we compare HZ and PHN incidence in the period 2006–2013 with our previous estimates from 2000–2006. We also examine time trends in HZ and PHN incidence and in health care resource utilisation over this period.

## Methods

### Incidence of herpes zoster and PHN

Trends in age specific incidence of herpes zoster in Australia were estimated using general practice and pharmaceutical prescription data as previously described [2].

#### Age specific zoster and PHN incidence from BEACH database

The BEACH database is a cross-sectional paper based data collection of a nationally representative sample of general practitioners (GPs) [31]. Approximately 250 GPs are surveyed each quarter across Australia. Each GP records details of 100 consecutive GP-patient encounters of all types. The participating GPs are randomly selected from the Medicare Australia (formerly the Health Insurance Commission) list of active GPs (those who provided more than 375 GP services in the previous quarter). Patient reasons for encounter, problems managed, therapeutic procedures, other non-pharmacological treatments, referrals, and pathology and imaging are classified using ICPC-2 (the International Classification of Primary Care)[32], but coded more specifically in an Australian general practice terminology ICPC-2 Plus [33]. The ICPC is regarded as the international standard for data classification in primary care. For the herpes zoster analysis we extracted all encounters involving management of herpes zoster (ICPC-2 Plus codeS70001) or shingles (S70003), and for PHN analysis those involving management of PHN (S70002).

We have previously published an analysis based on data collected from 6,460 GPs during a 78 months period from April 2000 to September 2006, providing a database of 646,000 encounters [2]. This study used all new data available, collected from 6,302 GPs during a 78 month period from October 2006 until March 2013 (630,200 encounters).

Individuals with herpes zoster are likely to consult a GP [34]. The consultation rate for new herpes zoster problems can therefore be assumed to provide a reasonable estimate of its incidence [34–37]. New problems are defined as those never managed by any doctor in this patient, or a first medical consultation for a new episode of a recurrent problem. Age specific herpes zoster and PHN incidence was measured by the annual rate of “new” herpes zoster problems (codes S70001 and S70004) or “new” PHN problems (code S70002) recorded in the BEACH database respectively, stratified by 10 year age bands (or 25 year age bands for the population aged less than 50 years), extrapolated to total average annual Medicare claimed GP attendances over the period, then divided by the population for Australia in each age group [38,39]. All estimates presented have been rounded up to the nearest 50.

The time trend in the rate of management of new zoster problems per 1,000 encounters from April 1998 to March 2013 was examined by linear regression analysis for all patients, patients aged less than 60 years and those aged 60 years and older. This analysis was conducted over the full period for which there is BEACH data available, using SAS software version 9.3 (SAS Institute, Cary, NC, USA) using procedures to adjust for the single-stage cluster sampling around GP. For the comparison of the two periods, significance difference was determined through non-overlapping 95% confidence intervals (CIs). Non-overlapping CIs are a more conservative measure of significance than the 5% level with p<0.01 [40]. This method reduces the chance of false positive results but increases the chance of false negative results.

#### Age specific zoster incidence from antiviral prescriptions

In Australia the Pharmaceutical Benefits Scheme (PBS) provides medicines to all Australian residents at a Government-subsidised price. The PBS Schedule lists three direct acting antiviral drugs for the treatment of patients with herpes zoster: aciclovir (PBS Item No. 01052J), famciclovir (08002E and 8897G) and valaciclovir hydrochloride (08064K). These PBS listed drugs are restricted to be used within 72 hours of the onset of the rash, and are thus representative of incident zoster cases.

Updated age specific annual antiviral prescription numbers were estimated from scripts supplied to a sample of the Australian population drawn from Pharmaceutical Benefits payment records from July 2006 to June 2012 (Department of Human Services, analysis provide by HI Connections Pty Ltd), for comparison with earlier published estimates derived from the July 2002 to June 2006 period. Results were validated against prescription data on the Medicare Australia website.

Annual antiviral prescription rates and average rates over the July 2002 to June 2006 and the July 2006 to June 2012 periods were estimated for specific age groups using the “ci” command in Stata version 11.2, with Poisson 95% confidence intervals and the Australian population in each age group defined as the exposure variable. To analyse time trends in age-specific rates of antiviral prescriptions, annual rates were analysed over the full 2002–03 to 2011–12 period by generalised linear modelling with a logarithmic link function. Goodness of fit of the models were checked using the Pearson statistic and the reported models fit the data well (p > 0.99).

To estimate zoster incidence based on antiviral prescriptions for herpes zoster, age specific numbers of antiviral prescriptions were adjusted for the estimated proportion of new zoster cases that were prescribed direct acting antivirals (ATC code J05A) during the corresponding periods, derived from analyses of the BEACH database. Age specific incidence rates over the July 2002 to June 2006 and the July 2006 to June 2012 periods were then estimated using the “ci” command in Stata version 11.2, with Poisson 95% confidence intervals and the Australian population in each age group defined as the exposure variable. For the comparison of the two periods, significance difference was determined through non-overlapping 95% confidence intervals (CIs). Non-overlapping CIs are a more conservative measure of significance than the 5% level with p<0.01 [40].

### Hospitalisations

The Australian Institute of Health and Welfare National Hospital Morbidity database contains demographic, diagnostic and length of stay information on episodes of care for patients admitted to Australian hospitals [41,42].

Age-specific numbers of hospital separations for patients with a principal or non-principal diagnosis of herpes zoster (ICD-10-AM code B02) were obtained from the National Hospital Morbidity Database for the July 2007 to June 2012 period, for comparison with the earlier July 1998 to June 2005 period. Data sources included on-line interactive principal diagnosis data cubes (2007) [41,42] and two unpublished commissioned reports also addressing hospitalisations with non-principal diagnosis of zoster. Annual data was only available for the July 2007 to June 2012 period.

Average rates over the July 1998 to June 2005 and the July 2007 to June 2012 periods were estimated for specific age groups using the “ci” command in Stata version 11.2, with Poisson 95% confidence intervals and population defined as the exposure variable. The age-specific time trends in yearly rates of hospitalisations between July 2007 and June 2012 were analysed by linear regression. Normal distribution of residuals was confirmed using the Shapiro-Wilk test.

### Emergency Department Visits

Age-specific numbers of admitted and non-admitted emergency department (ED) visits for zoster in New South Wales (NSW) were obtained for the July 1998 to June 2012 period from NSW Emergency Department Data Collection (SAPHaRI), Centre for Epidemiology and Evidence, NSW Ministry of Health. The analysis included the 45 hospitals that reported continuously to the NSW ED Data Collection (EDDC) over the entire period and that used one of three diagnosis classifications including the Systematised Nomenclature of Medicine Clinical Terminology (SNOMED CT), ICD-10 and ICD-9. These hospitals represent approximately two thirds of total ED activity in NSW and include most of the larger urban and regional hospitals in NSW. Total number of non-admitted ED visits was estimated by multiplying the reported non-admitted numbers by 3/2.

Age-specific numbers of admitted and non-admitted emergency department (ED) visits for zoster in Victoria were obtained for the July 2005 to June 2012 period from the Victorian Emergency Minimum Dataset (VEMD), provided by HOSdata, Information and Funding Systems Branch, Victoria. ED visits that documented the code B02.9 Zoster without complication, in any of the 3 diagnoses recorded in the database were extracted. No other zoster codes are provided for ED coding in Victoria.

Age specific rates of ED visits in NSW and Victoria were estimated for the July 2006 to June 12 period using the “ci” command in Stata version 11.2 with Poisson 95% confidence intervals and the NSW or Victorian population in each age group defined as the exposure variable, for comparison with the earlier July 1998 to June 2005 periods. Estimated resident NSW and Victoria population data was obtained from the Australian Bureau of Statistics [39].

The age-specific time trends in yearly rates of admitted and non-admitted ED visits over the full period of data availability were analysed by linear regression. Normal distribution of residuals was confirmed using the Shapiro-Wilk test.

### Ethics Statement

During the data collection period for this study the BEACH program was approved by the Human Research Ethics Committee of the University of Sydney and the Ethics Committee of the Australian Institute of Health and Welfare. Our method involves the collection of data from unidentifiable, consenting patients. A patient information card is supplied in the research kit, which GPs are instructed to show to patients in order to obtain informed consent (an example shown in Britt et al. [43]). If the patient chooses not to participate their encounter details are not recorded. GPs are asked not to provide written consent to the research body, as this prevents patients remaining anonymous. These methods comply with the Ethics requirements for the BEACH program.

All PBS, hospital and emergency department data was provided as unidentifiable, aggregate data.

## Results

### National age specific herpes zoster and PHN incidence based on extrapolated BEACH data

New cases of herpes zoster were managed at 655 GP encounters recorded in the BEACH database from October 2006 to March 2013 inclusive, extrapolating to an estimated national annual average of 122,960 new zoster cases and an estimated incidence of 5.6 per 1,000 persons (95% CIs: 5.2–6.1). This was an increase from 4.7 per 1,000 persons (95% CIs: 4.3–5.0) estimated from the BEACH data recorded in the earlier April 2000 to September 2006. As seen for the earlier period, the updated analysis demonstrated that zoster incidence increased with age, from 1.8 per 1,000 persons aged 0–24 years, to 19.9 per 1,000 for those aged 80 years and over (Table 1).

**Table 1:** National age specific incidence of herpes zoster and post herpetic neuralgia based on extrapolated BEACH data.

Age	BEACH database April 2000 – September 2006^1^				BEACH database October 2006 – March 2013			
	Population (2004)^2^	New zoster/PHN encounters recorded	Estimated new zoster/PHN cases (National annual estimates)^3^ (95% CI)	Estimated new zoster/PHN cases per 1,000 persons^4^ (95% CI)	Population (2007–12)^5^	New zoster/PHN encounters recorded	Estimated new zoster/PHN cases (National annual estimates)^6^ (95% CI)	Estimated new zoster/PHN cases per 1,000 persons^7^ (95% CI)
Herpes Zoster								
<25	6,741,520	61	9,391 (6,894 – 11,887)	1.39 (1.02 – 1.76)	7,187,000	69	12,740 (9,660 – 15,820)	1.77 (1.34 – 2.20)
25–49	7,314,664	163	25,093 (21,241 – 28,945)	3.43 (2.90 – 3.96)	7,747,655	149	27,510 (23,000 – 32,020)	3.55 (2.97 – 4.13)
50–59	2,525,527	107	16,450 (13,300–19,650)	6.52 (5.26 – 7.78)	2,756,079	94	17,350 (13,870 – 20,840)	6.30 (5.03 – 7.56)
**60–69**	**1,651,276**	**92**	**14,150 (11,200 – 17,150)**	**8.58 (6.78 – 10.37)**	**2,053,592**	**152**	**28,060 (23,600 – 32,530)**	**13.66 (11.49 – 15.84)**
70–79	1,178,105	111	17,100 (13,750 – 20,450)	14.50 (11.66 – 17.35)	1,253,830	104	19,200 (15,470 – 22,930)	15.31 (12.34– 18.29)
80+	680,412	69	10,600 (8,150 – 13,100)	15.61 (11.95 – 19.28)	807,600	87	16,060 (12,670 – 19,450)	19.89 (15.69– 24.08)
Post Herpetic Neuralgia								
<25	6,741,520	4	616 (12–1219)	0.09 (0–0.18)	7,187,000	1	180 (0–550)	0.03 (0–0.08)
25–49	7,314,664	3	461 (0–984)	0.06 (0–0.13)	7,747,655	8	1,480 (450–2500)	0.19 (0.06–0.32)
50–59	2,525,527	12	1,850 (800–2,900)	0.73 (0.32–1.15)	2,756,079	15	2,770 (1,370–4170)	1.01 (0.50–1.51)
60–69	1,651,276	13	2,000 (900–3,100)	1.21 (0.55–1.88)	2,053,592	23	4,250 (2,510–5,980)	2.07 (1.22–2.91)
70–79	1,178,105	18	2,750 (1,500–4,050)	2.35 (1.26–3.44)	1,253,830	23	4,250 (2,510–5,980)	3.39 (2.00–4.77)
80+	680,412	14	2,150 (1,050–3,300)	3.16 (1.54–4.85)	807,600	23	4,250 (2,510–5,980)	5.26 (3.11–7.40)

BEACH: Bettering the Evaluation and Care of Health; 95% CI: 95% Confidence interval. Bolded data in table indicates statistically significant results.

^1^. Data for the 50–59, 60–69, 70–79,80+ and 50+ age groups reproduced from Table 3 in (Stein et al 2009).

^2^. Australian Bureau of Statistics (ABS). Series B projections by age and sex. Australian Bureau of Statistics, 2007, available at http://www.abs.gov.au/AUSSTATS/abs@.nsf/DetailsPage/3222.02004%20to%202101?OpenDocument.

^3^. Based on the rate of encounters for new herpes zoster problems extrapolated to national annual average of 99.45 million general practice encounters.

^4^. Calculation based on the 2004 Australian population (reproduces the data from (Stein et al 2009).

^5^. Average estimated resident population from 2007 to 2012. Australian Bureau of Statistics (ABS). 3101.0—Australian Demographic Statistics, Jun 2013. 2013, available at http://www.abs.gov.au/ausstats/abs@.nsf/mf/3101.0.

^6^. Based on the rate of encounters for new herpes zoster or PHN problems extrapolated to national annual average of 116.35 million general practice encounters.

^7^. Calculation based on the average estimated resident population of Australia from 2007 to 2012.

Of new zoster cases occurring in the most recent period, 80,670 (67%) occurred among individuals aged 50 years and older, for whom the zoster vaccine is indicated, with an estimated incidence of 11.7 per 1,000 persons (95% CI: 10.6–12.9) in this age group. Most of the observed increase in incidence of herpes zoster was observed in the population aged 60 years and older, with an estimated incidence of 15.4 (95% CI: 13.8–17.0) per 1,000 persons, significantly above that of the earlier period, 11.9 (95% CI: 10.5–13.4) per 1,000 persons (p < 0.01).

This observation was confirmed by analysis of the trend in the rate of consultations for new zoster problems between 1998 and 2013. Overall, there was a significant increase in the management rate of new zoster cases at a rate of 2.5 per 100,000 patient encounters per year (p<0.0001). However this trend was twice as large for patients aged 60 years and older (3.1, p = 0.012) than it was for patients aged less than 60 years (1.5, p = 0.024).

**Fig 1:**
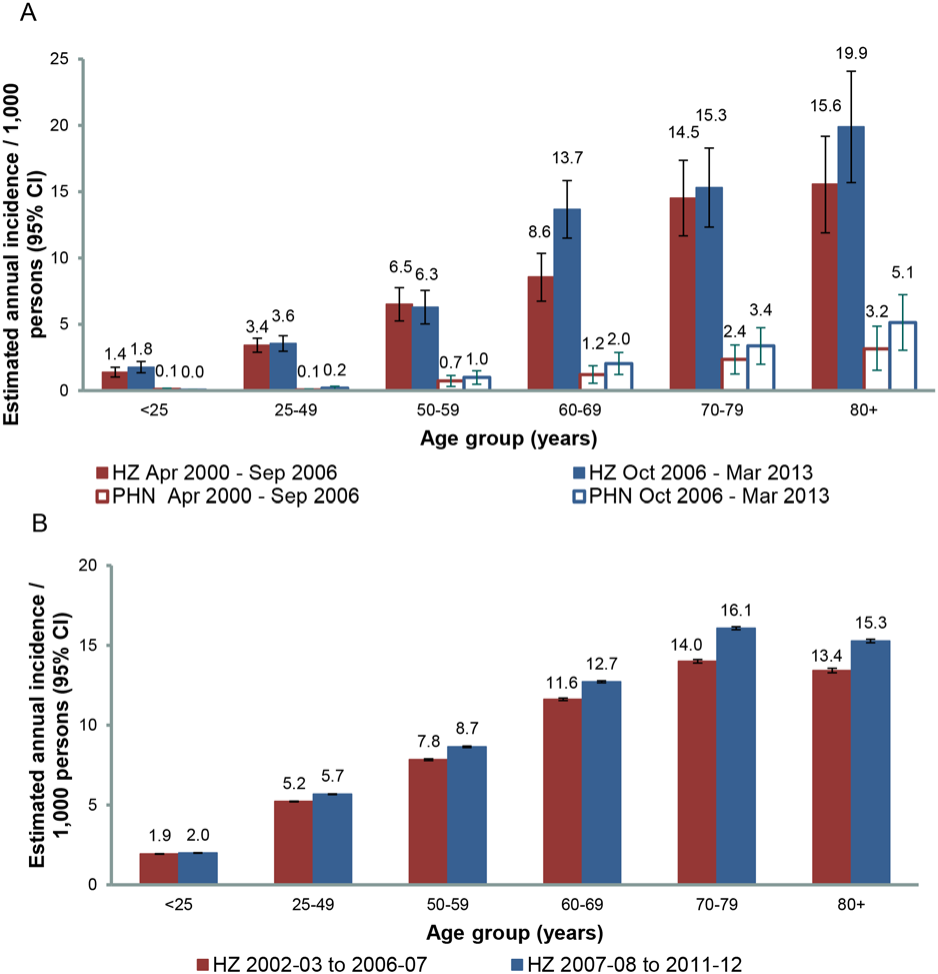
Age dependent incidence of herpes zoster and PHN over two consecutive time periods.

New cases of PHN were managed at 93 GP encounters recorded in the BEACH database between October 2006 until March 2013, extrapolating to an estimated annual average of 17,170 new PHN cases and an estimated incidence of 0.79 per 1,000 persons (95% CIs: 0.63–0.95), compared with 0.51 per 1,000 persons (95% CIs: 0.38–0.63) in the earlier period. PHN incidence increased with age, from 0.03 per 1,000 persons aged 0–24 years, to 5.3 per 1,000 for those aged 80 years and over (Table 1). In the population aged 60 years and older, PHN incidence was 3.10 (95% CI: 2.37–3.82) per 1,000 persons, compared with the 1.97 (95% CI: 1.40–2.55) incidence per 1,000 persons estimated for the earlier BEACH period, in line with the increased zoster incidence. Fig 1 shows the age dependent incidence of herpes zoster and PHN over two consecutive time periods, roughly corresponding respectively to the periods prior to and after the introduction of universal infant varicella vaccination in Australia.

### National age specific zoster incidence based on antiviral prescriptions

**Fig 2:**
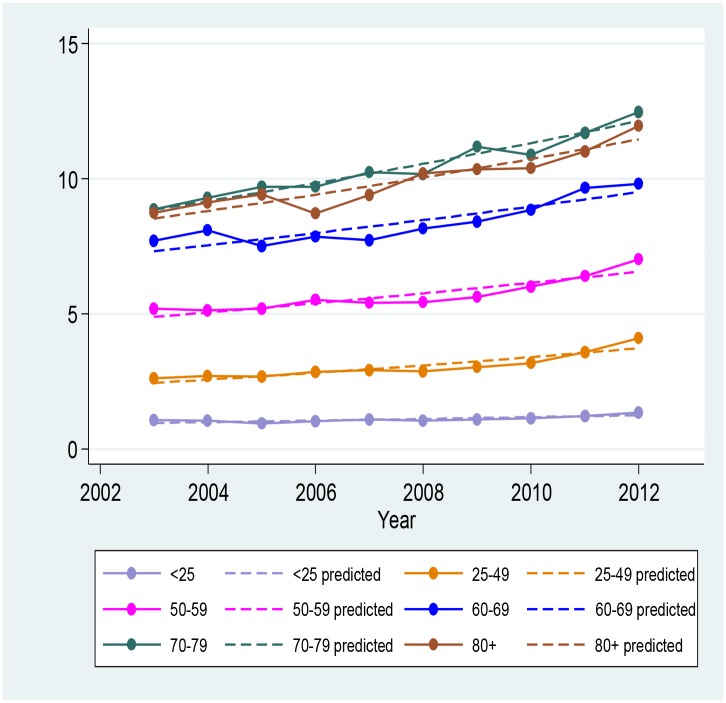
Trend in antiviral prescription rates over time in the Australian population.

The estimated annual numbers of antivirals prescribed for herpes zoster on the PBS increased from 69,775 (3.5 per 1,000 persons) during the July 2002 to June 2006 period to 90,858 (4.2 per 1,000 persons) during the July 2006 to June 2012 period. The rates of PBS antiviral prescriptions for herpes zoster increased with age, from 1.2 per 1,000 persons aged less than 25 years, to 11.1 and 10.6 per 1,000 persons for those aged 70 to 79 years and 80 years and over respectively during the more recent period (Table 2). Trend analyses (Fig 2) demonstrated that between July 2002 and June 2012, the overall rate of prescriptions of antivirals specific for the treatment of herpes zoster increased significantly by 4.2% per year (3.2–5.2%). In the population aged 60 years and older the estimated increase was 3.1% per year (2.4%- 3.8%) (p < 0.001 for both analyses). The age-specific observed and predicted rates of antiviral prescriptions per 1,000 persons are presented in Fig 2.

**Table 2:** National age specific incidence of herpes zoster based on analysis of PBS antiviral prescriptions for herpes zoster.

Age	July 2002 – June 2006				July 2006 – June 2012			
	Average Population^1^	Average Antiviral Prescriptions per year^2^	Estimated prescription rate per 1,000 persons (95% CI)^3^	Estimated zoster cases per 1,000 persons (95% CI)^4^	Average Population^1^	Average Antiviral Prescriptions per year^2^	Estimated prescription rate per 1,000 persons (95% CI)^3^	Estimated zoster cases per 1,000 persons (95% CI)^5^
**< 25**	**6,755,991**	**6,810**	**1.01 (1.00 – 1.02)**	**1.95 (1.93 – 1.96)**	**7,195,487**	**8,311**	**1.16 (1.15 – 1.17)**	**2.00 (1.99 – 2.01)**
**25 – 49**	**7,286,106**	**19,720**	**2.71 (2.69 – 2.73)**	**5.22 (5.20 – 5.25)**	**7,748,732**	**25,441**	**3.28 (3.27 – 3.30)**	**5.68 (5.66 – 5.70)**
**50 – 59**	**2,524,059**	**13,267**	**5.26 (5.21 – 5.30)**	**7.84 (7.79 – 7.90)**	**2,749,976**	**16,486**	**6.00 (5.96 – 6.03)**	**8.65 (8.61 – 8.70)**
**60 – 69**	**1,660,706**	**12,937**	**7.79 (7.72 – 7.86)**	**11.63 (11.54 – 11.71)**	**2,059,052**	**18,145**	**8.81 (8.76 – 8.86)**	**12.72 (12.65 – 12.78)**
**70 – 79**	**1,162,886**	**10,917**	**9.38 (9.30 – 9.47)**	**14.01 (13.90 – 14.12)**	**1,248,226**	**13,905**	**11.14 (11.06 – 11.22)**	**16.07 (15.98 – 16.16)**
**80+**	**680,569**	**6,122**	**9.00 (8.88 – 9.11)**	**13.43 (13.29 – 13.56)**	**809,216**	**8,568**	**10.59 (10.50 – 10.68)**	**15.28 (15.17 – 15.39)**

Bolded data in table indicates statistically significant results.

^1^. Average estimated resident population of Australia. Australian Bureau of Statistics (ABS). 3101.0—Australian Demographic Statistics, Jun 2013, available at http://www.abs.gov.au/ausstats/abs@.nsf/mf/3101.0; June 2003 to June 2012 populations were assigned to the 2002–03 to 2011–12 financial years respectively.

^2^. Prescription numbers estimated from scripts supplied to a sample of the Australian population drawn from PBS payment records by financial year from July 2002—June 2012. Note for the July 2002—June 2006 period there were very minor differences from estimates presented in (Stein et al 2009), reflecting changes in the updated dataset.

^3^. Rates estimated using the “ci” command in Stata version 11.2, with Poisson 95% confidence intervals and population defined as the exposure variable, reflecting the variability in annual numbers of antiviral prescriptions.

^4^. Extrapolated from annual antiviral prescriptions for herpes zoster recorded on the PBS, adjusted for the proportion of encounters for a new zoster problem in which antivirals are prescribed, derived from the BEACH database (51.8% and 67.0% for those aged less than 50 years or 50 years and older respectively). Rates estimated using the “ci” command in Stata version 11.2, with Poisson 95% confidence intervals and population defined as the exposure variable, reflecting the variability in annual numbers of antiviral prescriptions.

^5^. Extrapolated from annual antiviral prescriptions for herpes zoster recorded on the PBS, adjusted for the proportion of encounters for a new zoster problem in which antivirals are prescribed, derived from the BEACH database (57.8% and 69.3% for those aged less than 50 years or 50 years and older respectively). Rates estimated using the “ci” command in Stata version 11.2, with Poisson 95% confidence intervals and population defined as the exposure variable, reflecting the variability in annual numbers of antiviral prescriptions.

The increased rate of antiviral prescriptions was observed despite relatively stable prescriber behavior, with antivirals being prescribed by GPs in similar proportion of encounters for new zoster problems in the two periods examined (61.5% (57.6–65.4) between April 2000 and September 2006 and 65.6% (61.9–69.3) between October 2006 and March 2013) from BEACH data. This stable prescriber behaviour over time was observed for all patient age groups. However, antiviral prescriptions were less frequent in encounters for new zoster problems in patients younger than 50 years across the two periods (51.8% (45.0–58.5) and 57.8% (51.3–64.3)) compared those aged 50 years and older (67.0% (62.3–71.8) and 69.3% (64.9–73.8)). These age-group specific estimates were used to estimate incidence of herpes zoster.

Adjustment of the annual numbers of antivirals prescribed for herpes zoster cases by the estimated proportion of encounters in which such antivirals are prescribed results in an estimated average of 131,108 new zoster cases per year during the more recent July 2006 to June 2012 periods, corresponding to an annual incidence rate of 6.01 (95% CI: 6.0–6.02) per 1,000 persons. This had increased from 5.18 (95% CI: 5.17–5.20) per 1,000 persons estimated for the earlier July 2002 to June 2006 period (Table 2). As seen for the earlier period, the updated analysis demonstrated that zoster incidence increased with age, from 2.0 per 1,000 persons aged 0–24 years, to 16.1 per 1,000 for those aged 70 to 79 years and 15.3 per 1,000 for those aged 80 years and over (Table 2).

Of new zoster cases in the most recent period, 82,402 (63%) are estimated among individuals aged 50 years and older, for whom the zoster vaccine is indicated, with an estimated incidence of 12.0 per 1,000 persons (95% CI: 11.96–12.03) in this age group. In the population aged 60 years and older, the estimated incidence was 14.24 (95% CI: 14.19–14.29) per 1,000 persons, significantly above that of the earlier period, estimated as 12.77 (95% CI: 12.71–12.83) per 1,000 persons (p < 0.01).

### Hospitalisations

Between July 2007 and June 2012 there was a yearly average of 5,878 hospitalisations for herpes zoster, a rate of 26.7 (95% CI: 26.0–27.4) per 100,000 persons. This was increased from 4,827 in the earlier July 1998 to June 2005 period, corresponding to 24.6 (95% CI: 24.1–25.5) hospitalisations per 100,000 persons.

**Fig 3:**
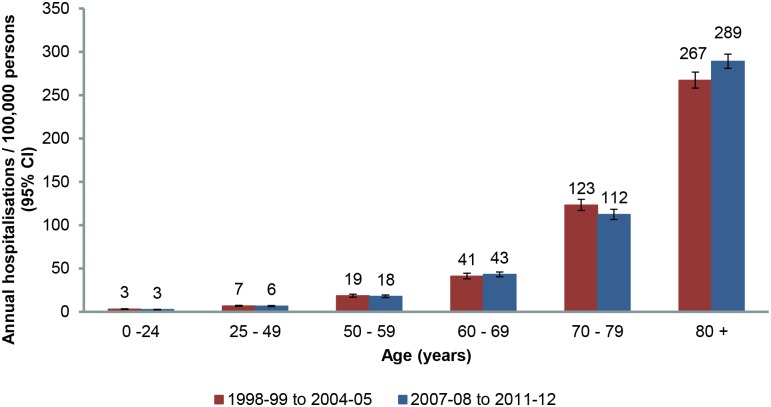
Estimated age specific hospitalisation rates for herpes zoster over two time periods.

**Fig 4:**
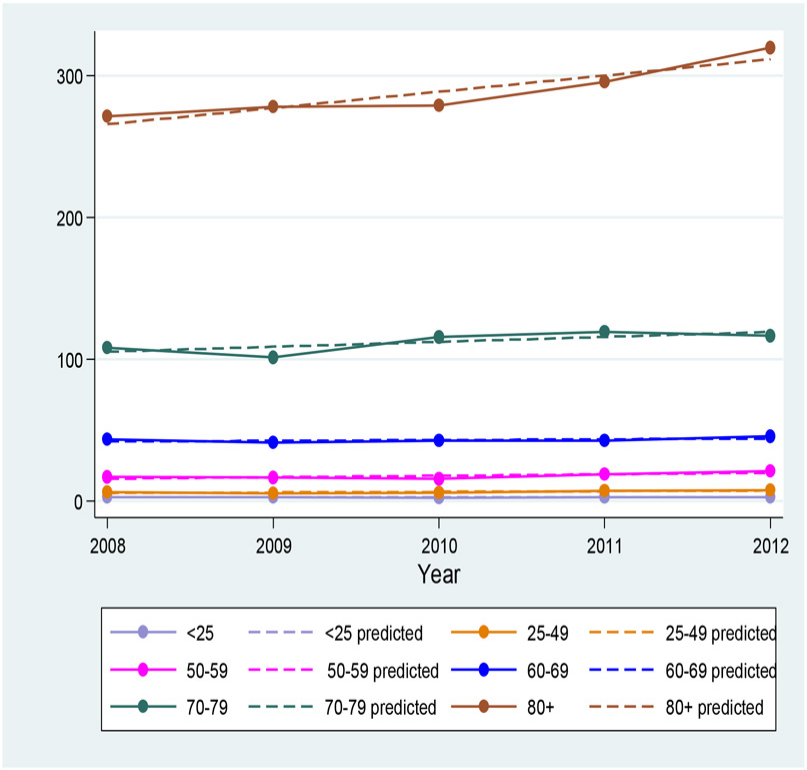
Trend in hospitalisation rates over time in the Australian population.

Hospitalisation rates increased with age (Fig 3). Age specific rates remained stable across the two periods for all age groups with the exception of rates in the population aged 80 years and older, which demonstrated a significant increase from 267.3 (95% CI: 258.2–276.6) to 289.1 (95% CI: 281.0–297.5) per 100,000 persons. Analysis of the trend in hospitalisation rates between 2008 and 2012 in this population confirmed this finding, as rates increased by 11.4 (95% CI: 3.1–19.8) hospitalisations per 100,000 persons per year (p = 0.023) (Fig 4).

### Emergency Department visits

#### New South Wales

Between July 2006 and June 2012 there was a yearly average of 2,184 emergency department visits for herpes zoster in New South Wales, a rate of 30.8 per 100,000 persons. This was increased from 1,474 in the earlier July 1998 to June 2006 period (rate of 22.4 per 100,000 persons). Most emergency department visits were not admitted into hospital.

**Fig 5:**
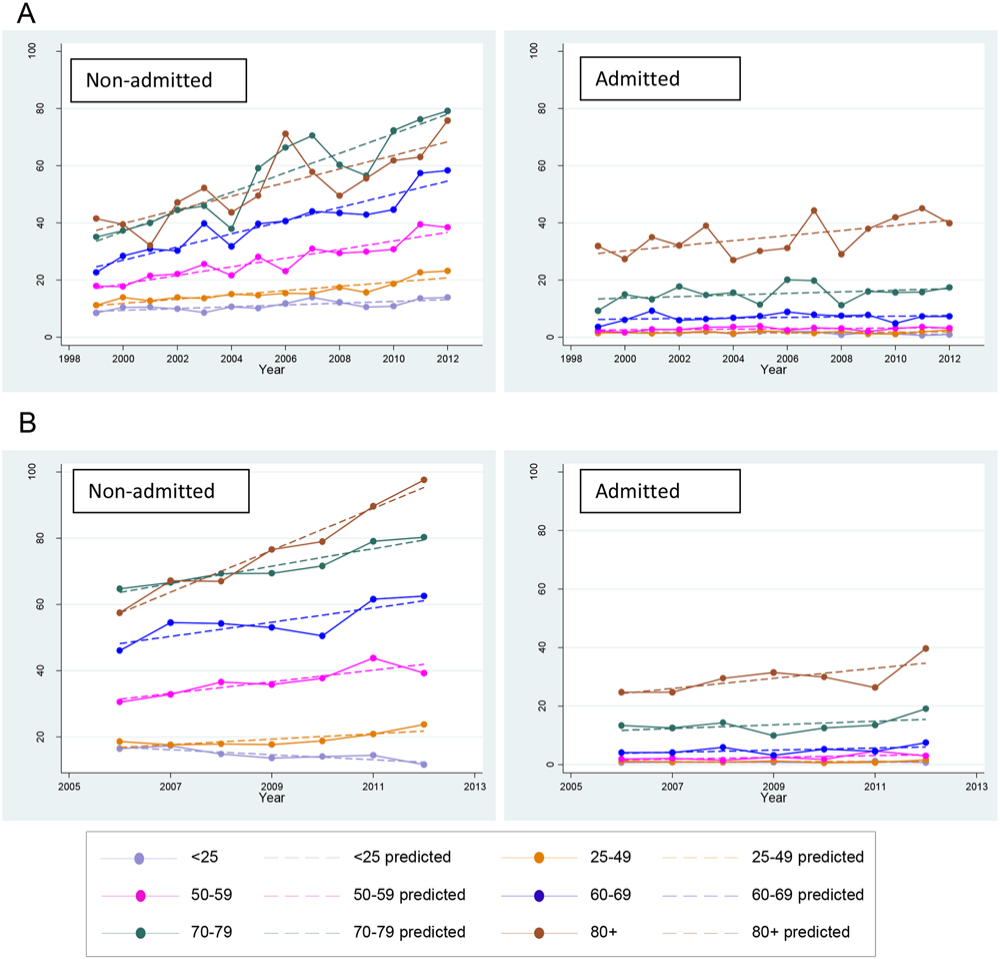
Trends in emergency department presentations for herpes zoster over time in NSW and Victoria.

The yearly average numbers of estimated non-admitted ED visits increased from 1,204 from July 1998 to June 2006 (18.3 (95% CI: 17.9–18.7) per 100,000 persons) to 1,856 between July 2006 and June 2012 (26.2 (95% CI: 25.7–26.7) per 100,000 persons). Trend analysis demonstrated a significant annual increase of 1.14 (95% CI: 0.91–1.37) non-admitted ED visits for zoster per 100,000 persons (p<0.001) per year between July 1998 and June 2012. The increasing trend was more pronounced with increasing age, with annual increases of 2.63 (95% CI: 2.02–3.25, p<0.001) visits per 100,000 persons aged 60 years and older (Fig 5).

Estimated admitted ED visits also increased, from 270 to 328 in the more recent period, with estimated rates of 4.1 (95% CI: 3.9–4.3) and 4.6 (95% CI: 4.4–4.8) per 100,000 persons respectively. However, this largely reflected a significant trend in the population aged 80 years and older, increasing by 0.89 (95% CI: 0.15–1.63) visits per 100,000 persons per year (p = 0.023), with other age groups remaining stable (Fig 5).

#### Victoria

Between July 2006 and June 2012 there was a yearly average of 1,750 emergency department visits for herpes zoster in Victoria, a rate of 32.4 per 100,000 persons, similar to that observed for the same period in NSW. Of these, 1,564 visits (29.0 per 100,000 persons) were not admitted into hospital, and 186 (3.5 per 100,000 persons) were admitted into hospital. The rate of ED visits increased with age (Fig 5).

Analysis of the trend in the rate of non-admitted ED visits between July 2005 and June 2012 demonstrated a significant annual increase of 0.97 (95% CI: 0.41–1.54) non-admitted ED visits for zoster per 100,000 persons (p = 0.007). The increasing trend was more pronounced with increasing age, with annual increases of 3.08 (95% CI: 1.74–4.42, p = 0.002) visits per 100,000 persons aged 60 years and older (Fig 5). In contrast, admitted ED visits remained generally stable (Fig 5).

## Discussion

This study provides strong evidence that the burden of HZ has increased in Australia over the last decade or more. Zoster incidence has been examined across two periods of time 2000 to 2006 and 2006 to 2012 using two different population based approaches in the BEACH GP encounter database and the PBS database of HZ antiviral prescriptions. These analyses give largely consistent estimates and a broadly similar overall increase in zoster incidence of 15–20%. The relatively lower zoster incidence estimated for persons aged 80 years and older in the HZ antiviral prescription analysis is likely a reflection of the unknown proportion of scripts provided to this population on the Repatriation Pharmaceutical Benefits Scheme, which are excluded from the analysis. Looking at the trend analysis of HZ antiviral prescriptions, the increase in zoster incidence is seen both pre- and post- universal varicella vaccination with no marked change in the rate of increase. The findings are supported by the increases in non-admitted emergency department visits over time in two states of Australia.

The increases in HZ and PHN incidence and in non-admitted emergency department visits are most prominent in the 60 years and older population. In contrast, the hospitalisation data did not show increasing trends over time, except for the age group ≥80 years. The BEACH, prescribing and emergency data may be more sensitive data sets for monitoring HZ trends, particularly given that we have shown that most HZ encounters are not hospitalised.

The findings from our study are similar to previously published studies in both Australia and the USA. In their studies, Leung et al. and Jardine et al. demonstrated significant increases in herpes zoster rates particularly among the elderly, with the increase preceding the introduction of VZV vaccination [12,21]. Other Australian studies examining varicella and HZ trends from the mid 1990 demonstrated similar increases in community based consultations for zoster, a decrease in varicella hospitalisation rates and a significant increase in HZ over time [14,24,44].

The factors underpinning the increase in HZ burden are not clear. Consistent with the predictions of modeling studies, [17,18], there is increasing evidence supporting the Hope-Simpson hypothesis and that the impact of VZV vaccination may at least in part explain the observed rise in incidence of HZ [45–48]. However, the current study as well as previous studies show that at least some of the increase in disease burden precedes the introduction of VZV vaccination [12,49,50]. The reasons for the pre-vaccination increase are likely to be complex and multi-faceted, including increased use of immunsuppressive therapies in medical care.

Whatever the underlying causes of the increase in zoster incidence, the morbidity associated with zoster and PHN continues to increase with age consistent with the decline in immunity with ageing. The latter is evident in the progressively increasing incidence of PHN, the most common complication of HZ. PHN is characterised by clinically significant pain which may persist for months or years after rash onset, is significantly difficult to treat and is associated with a significant decline in quality of life [51–53]. These factors make a strong case for consideration of the zoster vaccine. Decisions around vaccination are complicated by evidence of decreasing efficacy against zoster incidence based on age at vaccination [27] and waning in efficacy against zoster burden based on time since vaccination [30]. Waning of vaccine-induced immunity occurs with most vaccines and reflects the natural phenomenon of immunosenescence [54]. Waning of vaccine-induced immunity is critically important for infant vaccination, who have a full lifespan ahead of them, but is less important for the elderly, when expected lifespan is short. The value of vaccines is a function of both immunogenicity and disease burden, and elderly vaccines should be considered within this equation [54]. Despite lower efficacy with age, HZ vaccination may have value in the older elderly because of higher disease burden [54]. Given waning vaccine immunity and limited duration of immunity, however, the timing and age of vaccination should be carefully planned to ensure that disease burden is not shifted further into unvaccinated older age groups, who have higher incidence of HZ and PHN. The efficacy of the HZ vaccine against PHN, however, is not age dependent [27]. This is the most debilitating complication of HZ, and prevention of PHN is a worthy goal of vaccination.

The limitations of this study relate to the data sources use. The BEACH database relies on accurate classification of encounters into those related to new versus old zoster problems, and the relatively low frequency of zoster management results in wide confidence intervals around the estimates. Furthermore, the analysis does not capture zoster cases treated in hospitals by specialists without a visit to a GP. The main limitation of the antiviral prescription data is that estimates of zoster incidence require adjustment for the proportion of encounters for new zoster problems in which antivirals are prescribed. These are derived from analysis of the BEACH database, and are thus subject to the limitations described above. Of note, there has been little progress made in improving the proportion of people receiving antivirals for zoster, especially in the >50 group, despite considerable efforts to do so, suggesting patients are probably not presenting to the GP early enough. A further limitation is that the PBS dataset used does not capture prescriptions provided under the Repatriation Pharmaceutical Benefits Scheme, which could represent approximately 7% of prescriptions in those aged 60 years and older, based on the proportion of zoster patients reported to hold a Veterans’ Affairs card in the BEACH database. Limitations of the ED visit data include mixed diagnosis classifications and substantial variation in diagnosis reporting completeness over time at some of the included hospitals. In addition, some of the SNOMED CT codes have ambiguous descriptions that could have led to inclusion of varicella infections in the analyses. However, the frequency of these codes was relatively small.

The main strengths of this study are that it compares zoster incidence across two periods of time which broadly correspond to the pre and post universal VZV vaccination periods in a well-defined population, using two different approaches that yield consistent estimates of zoster incidence, and that the significant increases in zoster incidence observed for the later period are supported by significant trends over time. A strength of this study is the range of data sources used. These data, particularly emergency department and HZ antiviral prescribing data, can be used for surveillance to monitor ongoing trends in HZ burden and the impact of any vaccination program.

Australia, like most developed countries has an ageing population [54] so healthy ageing and use of available preventive strategies are important. This study shows that the burden of HZ and PHN in the older Australian population is substantial and has been increasing since at least 2000. This increasing health burden justifies consideration of HZ vaccination for the elderly.
