# Correction: Increasing Trends of Herpes Zoster in Australia

**DOI:** 10.1371/journal.pone.0129872

**Published:** 2015-06-03

**Authors:** C. Raina MacIntyre, Alicia Stein, Christopher Harrison, Helena Britt, Abela Mahimbo, Anthony Cunningham

The first author’s name is spelled incorrectly. The correct name is: C. Raina MacIntyre. The correct citation is: MacIntyre CR, Stein A, Harrison C, Britt H, Mahimbo A, Cunningham A (2015) Increasing Trends of Herpes Zoster in Australia. PLoS ONE 10(4): e0125025. doi:10.1371/journal.pone.0125025
